# Including *α*_*s1*_*casein* gene information in genomic evaluations of French dairy goats

**DOI:** 10.1186/s12711-016-0233-x

**Published:** 2016-08-04

**Authors:** Céline Carillier-Jacquin, Hélène Larroque, Christèle Robert-Granié

**Affiliations:** GenPhySE, INRA, INPT, ENVT, Université de Toulouse, 31326 Castanet-Tolosan, France

## Abstract

**Background:**

Genomic best linear unbiased prediction methods assume that all markers explain the same fraction of the genetic variance and do not account effectively for genes with major effects such as the *α*_*s1*_*casein* polymorphism in dairy goats. In this study, we investigated methods to include the available *α*_*s1*_*casein* genotype effect in genomic evaluations of French dairy goats.

**Methods:**

First, the *α*_*s1*_*casein* genotype was included as a fixed effect in genomic evaluation models based only on bucks that were genotyped at the *α*_*s1*_*casein* locus. Less than 1 % of the females with phenotypes were genotyped at the *α*_*s1*_*casein* gene. Thus, to incorporate these female phenotypes in the genomic evaluation, two methods that allowed for this large number of missing *α*_*s1*_*casein* genotypes were investigated. Probabilities for each possible *α*_*s1*_*casein* genotype were first estimated for each female of unknown genotype based on iterative peeling equations. The second method is based on a multiallelic gene content approach. For each model tested, we used three datasets each divided into a training and a validation set: (1) two-breed population (Alpine + Saanen), (2) Alpine population, and (3) Saanen population.

**Results:**

The *α*_*s1*_*casein* genotype had a significant effect on milk yield, fat content and protein content. Including an *α*_*s1*_*casein* effect in genetic and genomic evaluations based only on male known *α*_*s1*_*casein* genotypes improved accuracies (from 6 to 27 %). In genomic evaluations based on all female phenotypes, the gene content approach performed better than the other tested methods but the improvement in accuracy was only slightly better (from 1 to 14 %) than that of a genomic model without the *α*_*s1*_*casein* effect.

**Conclusions:**

Including the *α*_*s1*_*casein* effect in a genomic evaluation model for French dairy goats is possible and useful to improve accuracy. Difficulties in predicting the genotypes for ungenotyped animals limited the improvement in accuracy of the obtained estimated breeding values.

## Background

With the recent development of molecular technologies, genomic selection is now used for several species, and major genes can be identified and sequenced. Selection for specific alleles of several major genes is already implemented, such as the *prion protein* (*PrP*) gene for scrapie resistance in dairy sheep and goats [1, 2]. The genomic best linear unbiased prediction (GBLUP) approach is based on the assumption that many quantitative trait loci (QTL), each with a small effect, contribute to genetic variation [3, 4]. This assumption is violated for QTL with large effects [5, 6]. However, other methods, such as the Bayesian method, are able to consider that single nucleotide polymorphisms (SNPs) explain different proportions of the genetic variance. When genes with a large effect segregate, such as the *diacylglycerol O*-*acyltransferase 1* (*DGAT1*) gene for fat content in dairy cattle, these methods could outperform GBLUP in terms of accuracy [7–9]. These methods (Bayesian or GBLUP) are based on fitting single SNPs independently, but the effects of the alleles of some major genes, which result from an insertion or a deletion of several nucleotides are not completely captured by a single SNP (as for the *α*_*s1*_-*casein* polymorphism, Gwenola Tosser-Klopp, INRA, Toulouse, personal communication). Using haplotypes instead of single SNPs in a genomic evaluation model was proposed to model the effect of such multiallelic major genes but the number of effects to estimate was considerably larger than with the single SNP model [10–12] and thus was quite expensive in computing time. The marker-assisted selection based on QTL that is implemented in French dairy cattle breeds since 2001 could be an alternate solution. The largest QTL were selected using linkage disequilibrium and linkage analysis and the others were selected using the elastic-Net approach [5]. QTL effects were included in the genomic evaluations by considering that the effects of the SNP haplotypes were random [12, 13]. This approach in French dairy cattle slightly improved the accuracy of GEBV compared with classical GBLUP [13]. However it required several steps: the first steps are aimed at detecting QTL for each trait of interest and a further step for genomic evaluation. If information on the major gene with a complex polymorphism is available as well as information based on SNP data, it could be simpler to include this information in a GBLUP model in order to improve the accuracy of genomic estimated breeding values (GEBV). Indeed, if information on QTL haplotypes or major genes is available for all the animals in a population, it can be easily included in genomic evaluations. For dairy species for which females are not usually genotyped, genomic evaluations are based on daughter yield deviations (DYD) [5, 12, 14]. However, evaluations based on phenotypes of all individuals improve genomic accuracy [15]. Thus, to include the effect of a major gene in genomic evaluations, the missing genotypes need to be accounted for, which can be done by calculating for each animal the probabilities of carrying each possible genotype. These probabilities can be estimated based on iterative peeling methods which use animals with known genotypes and pedigree relationships [16, 17]. The gene content method is another approach that allows estimation of breeding values of ungenotyped animals by taking *α*_*s1*_-*casein* information on genotyped animals into account. This method uses a multiple trait model that considers information on production traits and the number of copies of a particular allele for genotyped animals related to ungenotyped individuals [18]. However, the concept of gene content was developed for biallelic loci and needs to be extended for a multiallelic situation as in the case of the *α*_*s1*_-*casein* polymorphism.

In French dairy goats, accuracy of genomic selection is not as high as in dairy cattle [19] owing to the size and structure of the reference population [20]. Genomic selection in French dairy goats uses the GBLUP approach, but higher accuracy is expected by implementing approaches that include well-known major genes such as the *α*_*s1*_*casein* gene. In dairy goats, different alleles of the *α*_*s1*_-*casein* gene have various effects on protein content, protein yield, milk yield [21] and fat content [22, 23]. Polymorphism of the caprine *α*_*s1*_-*casein* gene is one of the key factors that determine the technological properties of milk, such as cheese yield and cheese curd formation [24]. In the French dairy goat breeding scheme, all candidate bucks for progeny testing that were born after 1986 were genotyped at the *casein α*_*s1*_ gene using allele-specific polymerase chain reaction (PCR) and restriction fragment length polymorphism (RFLP) [25]. These genotypes were used to shortlist young candidates and eliminate males that carried alleles with a negative effect on protein content. To date, the effect of the genotype at the *casein α*_*s1*_ locus has not been included in the genetic evaluation of French Alpine and Saanen goats [26].

The goat *α*_*s1*_*casein* gene is a complex gene with at least 17 alleles: alleles *A*, *B*_*1*_, *B*_*2*_, *B*_*3*_, *B*_*4*_, *C*, *H*, *L* and *M* are associated with increased levels of *α*_*s1*_*casein* in the milk, alleles *E* and *I* with intermediate levels, alleles *D*, *F* and *G* with reduced levels, and alleles *O*_*1*_, *O*_*2*_, and *N* have no effect i.e. are null alleles [27, 28]. Alleles *A*, *B*, *C*, *E* and *F* were identified in French dairy goat populations with alleles *E* and *F* predominating in the Alpine and Saanen breeds, before 2000 [24]. The complex allelic variation in the *α*_*s1*_-*casein* gene cannot be captured by a single SNP, especially since some alleles, e.g. allele *E,* are characterized by the insertion of several nucleotides [29]. Moreover, after quality control, the number of available SNPs within the *α*_*s1*_*casein* gene region is limited.

The aim of this study was to compare accuracies of GEBV obtained with various genomic evaluation methods that include the effect of the genotype at the *α*_*s1*_*casein* gene as fixed or as random based on the available genotyping data for the *α*_*s1*_*casein* gene, in purebred or multi-breed genomic evaluations. First, we undertook a detailed description of the allele frequencies of the *α*_*s1*_*casein* gene in the French population and the effects of each *α*_*s1*_*casein* genotype on all traits that are under selection in dairy goats. Then, we tested the impact of including the effect of a known *α*_*s1*_*casein* genotype in the genomic evaluation based on the daughter yield deviations (DYD) of the males [14]. We tested two methods that take the effect of the *α*_*s1*_*casein* genotype into account in genomic evaluation models based on all females with phenotypes (single-step model [30]).

## Methods

### Data

We used SNP genotypes for 470 Alpine and 353 Saanen males that had been previously obtained with the Illumina goat SNP50 BeadChip [31] as described in [20] (Table 1). After a quality control that was done separately for each breed and was based on the following criteria: a minor allele frequency (MAF) higher than 1 %, a call rate higher than 98 % and a call frequency higher 99 %, 46,959 validated SNPs remained for analysis.

**Table 1:** Number of animals with information on the *α*_*s1*_ *casein* genotype and SNP50 k genotypes, and number of females with recorded performance and males with DYD

	Breed	Animals with *α*_*s1*_ *casein* genotype	Animals with SNP50 k genotype	Females with phenotypes	Males with DYD
Females	Alpine	1529	–	1,160,213	–
	Saanen	1420	–	1,511,991	–
Males	Alpine	1912	470	–	1912
	Saanen	1415	353	–	1415

For a much larger group of animals, RFLP and PCR techniques on blood DNA samples were applied to determine the genotypes at the *α*_*s1*_*casein* locus. Genotypes consisted of 19 pairs of the six alleles *A*, *B*, *C*, *E*, *F* and *O* (Table 2) from the 21 possible pairs since *α*_*s1*_*casein* genotypes *FO* and *OO* have never been observed in the French dairy goat population. All incomplete genotypes (20 % in this study), e.g. with one missing allele, were ignored. *α*_*s1*_*casein* genotypes were available for 6276 animals (Table 1) born between 1982 and 2011 that comprised 2949 females (1529 Alpine and 1420 Saanen) and 3327 males (1912 Alpine and 1415 Saanen) including the 823 males for which 50 k SNP genotypes were available.

**Table 2:** Effect of the *α*_*s1*_ *casein* genotype on protein content (g/kg) for a progeny-tested male population and estimated separately for the Saanen and Alpine breeds

Genotype group	*α*_*s1*_ *casein* genotype	Saanen	Alpine
Strong	*BC*	3.7	*
	*AB*	2.5	1.7
	*BB*	2.4	*
	*AA*	2.2	2.5
	*AC*	1.6	*
	*CC*	*	*
Intermediate	*CE*	1.0	*
	*AE*	1.0	1.0
	*BF*	0.6	0.9
	*CF*	0.6	*
	*BE*	0.5	1.1
	*AF*	0.5	0.7
	*AO*	*	*
	*BO*	*	*
	*CO*	*	*
Weak	*EE*	−0.7	0.2
	*EF*	−0.9	−0.4
	*EO*	*	*
	*FF*	*	*

* Effect was not estimated because no animals were recorded

Five production traits were analyzed: milk yield (kg), fat and protein yields (kg) and fat and protein contents (g/kg) as described in [19, 20]. We used variance components for estimation of breeding values as described in [19]. The phenotypes recorded on females, the weights given to phenotypes (defined according to lactation number and length of lactation) [19] and the pedigree used in the single-step model were from the official genetic evaluation of January 2013 that included 4,178,315 Alpine and 3,173,516 Saanen lactations from 1,160,213 Alpine and 1,511,991 Saanen females, respectively (Table 1), and 2,981,809 individuals that were used to construct the relationship matrix [19]. Based on the same official genetic evaluation, daughter yield deviations (DYD) i.e. average performances of the daughters corrected for environmental effects and merit of the dam were computed and used as male phenotypes. DYD were obtained from female phenotypes as described previously in [20], for the progeny-tested 1912 Alpine and 1415 Saanen bucks (Table 1). These DYD were weighted by effective daughter contributions [32] estimated from the official genetic evaluation of January 2013.

### Prediction of female genotypes at the *α*_*s1*_*casein* locus

The probabilities of each possible genotype at the *α*_*s1*_*casein* locus for all the females with phenotypes, that had not been genotyped (152,554 Alpine and 126,738 Saanen goats), were obtained separately for each breed based on the iterative peeling procedure implemented in the software developed by Vitezica [16, 17]. Iterations were stopped when the summed absolute difference in genotype probabilities between two iterations were less than 10^−3^ for all females and for each genotype probability. For computational reasons, predictions were made separately for three groups of animals per breed, i.e. animals born before 2000, born between 2000 and 2007, and born after 2007. The genotype probabilities were computed using data on all available *α*_*s1*_*casein* genotypes (i.e. 6276 animals, Table 1) and a simplified pedigree going back 2–7 generations depending on the individual.

### Estimation of the effects of *α*_*s1*_*casein* genotypes based on DYD phenotypes

The significance of the effect of each *α*_*s1*_*casein* genotype on the five milk production traits was tested on the combined dataset (Alpine + Saanen) and separately on the dataset for each breed by using analysis of variance in the GLM procedure of the SAS^®^ software. For this analysis, a simple model was used where DYD were explained only by a *α*_*s1*_*casein* genotype effect and a breed effect for the combined dataset. Genotypes with less than 10 animals were excluded from this analysis. The amount of DYD phenotypic variance explained by the *α*_*s1*_*casein* genotype was estimated by a restricted maximum likelihood (REML) algorithm implemented in the remlf90 software [33]. Estimations were obtained for the same populations as above (Alpine + Saanen, and Alpine and Saanen, separately) for the five traits using a simplified pedigree of 37,669 individuals going back 10 generations from the genotyped males. We used the following model:

1 $${\mathbf{DYD}} = {\mathbf{X}}{\varvec{\upbeta}} + {\mathbf{Zu}} + {\mathbf{Ts}} + {\mathbf{e}},$$

where ${\mathbf{e}}$ is a vector of random normal errors, ${\mathbf{DYD}}$ is a vector of the 3327 males DYD (Table 1) weighted by effective daughter contributions and ${\mathbf{X}}$ is an incidence matrix for the fixed effects $\left( {\varvec{\upbeta}} \right)$, which consisted of a mean effect or breed effects. ${\mathbf{u}}$ is a vector of breeding values considered as random effects such that ${\text{Var}}\left( {\mathbf{u}} \right) = {\mathbf{A}}\upsigma_{\text{u}}^{2}$ with ${\mathbf{A}}$ being the pedigree-based relationship matrix. The *α*_*s1*_*casein* genotype (s) was (were) considered as a normally distributed random effect(s) $N\left( {0,\upsigma_{\text{s}}^{2} {\mathbf{I}}} \right)$. ${\mathbf{T}}$ was an incidence matrix that related individuals to the effects of the 19 possible *α*_*s1*_*casein* genotypes (combination of the six alleles). Based on the estimated effects of the *α*_*s1*_*casein* genotypes on protein content, genotypes were classified into three groups according to their effect: strong i.e. more than 1.5 g/kg, intermediate i.e. between 0.5 and 1.5 g/kg and weak i.e. less than 0.5 g/kg, in order to simplify the models that used the predicted genotypes obtained with the iterative peeling approach.

### Models of genomic evaluation used

#### Analyses based on DYD phenotypes

First, we investigated how the effect of the *α*_*s1*_*casein* genotype could be integrated in genomic evaluations based on DYD for production traits and considering only the 3327 genotyped males [1912 Alpine and 1415 Saanen (Table 1)]. Four types of breeding values were analyzed:

1. Breeding values estimated based on pedigree information;
2. Breeding values estimated based on pedigree information and considering the *α*_*s1*_*casein* genotype as a fixed effect;
3. Breeding values estimated based on genomic information;
4. Breeding values estimated based on genomic information and considering the *α*_*s1*_*casein* genotype as a fixed effect.

These analyses were carried out on a multi-breed (Alpine + Saanen) dataset because the effects of *α*_*s1*_*casein* genotypes on milk production traits seem to be similar in both breeds (see “Effect of *α*_*s1*_*casein* genotypes on traits selected for in French dairy goats” section) and the official genetic evaluation is a multi-breed evaluation since variance components are similar for both breeds [34]. Analyses were based on the following model:

2 $${\mathbf{DYD}} = {\mathbf{X}}{\varvec{\upbeta}} + {\mathbf{Zu}} + {\mathbf{e}},$$

where, as in Model (1), ${\mathbf{X}}$ is an incidence matrix for the fixed effects ${\varvec{\upbeta}}$ (i.e. breed, mean and with or without an effect for *α*_*s1*_*casein* genotype) and ${\mathbf{u}}$ is a vector of the breeding values considered as random effects. Breeding values were estimated either from pedigree information only with ${\text{Var}}\left( {\mathbf{u}} \right) = {\mathbf{A}}\upsigma_{\text{u}}^{2}$ or from both genomic (50 k SNP) and pedigree information with ${\text{Var}}\left( {\mathbf{u}} \right) = {\mathbf{G}}\upsigma_{\text{u}}^{2}$, ${\mathbf{G}}$ being derived as follows [15]:

$${\mathbf{G}} = 0.95 \times \frac{{{\mathbf{MM^{\prime}}}}}{{2\mathop \sum \nolimits_{j = 1}^{p} q_{j} \left( {1 - q_{j} } \right)}} + 0.05 \times {\mathbf{A}},$$

where $p$ corresponds to the number of SNPs considered, $q_{j}$ is the estimated frequency of an allele at SNP $j$, and ${\mathbf{M}}$ is a centered incidence matrix of SNP genotypes [14]. The relevance of adding the *α*_*s1*_*casein* genotype, as a fixed effect, in genetic (based on pedigree information) or genomic (based on pedigree and 50 K SNP chip data) evaluations based on DYD was compared to models that do not include the effects of *α*_*s1*_*casein* genotypes. In all cases, genetic and genomic breeding values of animals were estimated with the BLUP model using blupf90 software [33].

#### Single-step model based on female phenotypes

The last part of this study consisted in including the effect of the *α*_*s1*_*casein* genotype in one-step genomic evaluations for protein content based on recorded females from official genetic evaluations. Since the *α*_*s1*_*casein* genotype has a clear effect on protein content, only this trait was considered here. In a first approach, we used genotype probabilities at the *α*_*s1*_*casein* locus that were computed as explained in “Prediction of female genotypes at the *α*_*s1*_*casein* locus” section. Two models were tested for each of the three datasets: (1) both breeds (Alpine + Saanen), (2) Saanen, and (3) Alpine goats, only. The first model was as follows:

3 $${\mathbf{y}}_{1} = {\mathbf{X}}_{1} {\varvec{\upbeta}}_{1} + {\mathbf{Z}}_{1} {\mathbf{u}}_{1} + {\mathbf{W}}_{1} {\mathbf{p}}_{1} + {\mathbf{T}}_{1} {\mathbf{s}}_{1} + {\mathbf{e}}_{1} ,$$

where ${\mathbf{y}}_{1}$ corresponds to the response vector for protein content for: (1) 7,351,831 Alpine and Saanen records, (2) 3,173,516 Saanen records only, or (3) 4,178,315 Alpine records only. The following fixed effects $\left( {{\varvec{\upbeta}}_{1} } \right)$ were considered: herd within year (33 years, from 1980 to 2013) and parity (i.e. 1, 2 and ≥3), age (30 levels: from 1 to 9 years) and month at delivery within year and area (four areas in France depending on goat breeding management), length (10 levels) of dry period within year and area, and breed (Alpine and Saanen for the two-breed population). ${\mathbf{X}}_{1}$ is an incidence matrix relating these fixed effects to observations. ${\mathbf{W}}_{1}$ is an incidence matrix relating the random animal permanent environmental effects $\left( {{\mathbf{p}}_{1} } \right)$ to observations, with ${\mathbf{p}}_{1}$ assumed to follow a multivariate normal distribution i.e. ${\mathbf{p}}_{1} \sim N\left( {\mathbf{0},{\mathbf{I}}_{t} \upsigma_{p}^{2} } \right)$. The vector ${\mathbf{s}}_{1}$ of probabilities of allele combinations, for the 19 genotypes (3553 levels), was also assumed to follow a multivariate normal distribution, i.e. ${\mathbf{s}}_{1} \sim N\left( {\mathbf{0},{\mathbf{I}}_{{{\text{s}}_{1} }} \upsigma_{{{\text{s}}_{1} }}^{2} } \right)$. ${\mathbf{T}}_{1}$ is an incidence matrix relating the elements of ${\mathbf{s}}_{1}$ to observations in ${\mathbf{y}}_{1}$. The vector ${\mathbf{e}}_{1}$ is an error term assumed to be normally distributed i.e. ${\mathbf{e}}_{1} \sim N\left( {\mathbf{0},\upsigma_{{{\text{e}}_{1} }}^{2} {\mathbf{I}}_{\text{v}} } \right)$. ${\mathbf{Z}}_{1}$ is an incidence matrix relating observations to normally distributed breeding values $\left( {{\mathbf{u}}_{1} } \right)$ with ${\text{Var}}\left( {{\mathbf{u}}_{1} } \right) = {\mathbf{H}}\upsigma_{{{\text{u}}_{1} }}^{2}$, where matrix ${\mathbf{H}}$ combines pedigree and genomic (50 k SNP chip) information as derived in [30]. Variance components for this model were estimated by the REML algorithm using remlf90 software [33].

For the second model tested, the 19 possible genotypes were divided into three groups according to their estimated effects on protein content obtained from REML estimations as described above (cf. “Estimation of the effects of *α*_*s1*_*casein* genotypes based on DYD phenotypes” section): group 1 included the genotypes that increased protein content by more than 1.5 g/kg (*AA*, *AB*, *AC*, *BB* and *BC*), group 2 those that increased protein content from 0.5 to 1.5 g/kg (*AO*, *AE*, *AF*, *BO*, *BE*, *BF*, *CO*, *CE* and *CF*) and group 3 those that increased protein content by less than 0.5 g/kg (*FF*, *FO*, *EE*, *EF* and *EO*). The following model used was:

4 $${\mathbf{y}}_{1} = {\mathbf{X}}_{1} {\varvec{\upbeta}}_{1} + {\mathbf{X}}_{2} \left( {{\mathbf{t}}_{g1} {\text{s}}_{g1} + {\mathbf{t}}_{{{\text{g}}2}} {\text{s}}_{g2} + {\mathbf{t}}_{g3} {\text{s}}_{g3} } \right) + {\mathbf{Z}}_{1} {\mathbf{u}}_{1} + {\mathbf{W}}_{1} {\mathbf{p}}_{1} + {\mathbf{e}}_{1} ,$$

with the same fixed $\left( {{\varvec{\upbeta}}_{1} } \right)$ and random permanent environmental $\left( {{\mathbf{p}}_{1} } \right)$ effects and the same vector of breeding values $\left( {{\mathbf{u}}_{1} } \right)$ as described in Model (3). In this model, ${\text{s}}_{g1}$, ${\text{s}}_{g2}$ and ${\text{s}}_{g3}$, correspond each to the fixed effect of the first (strong effect), the second (intermediate effect) and the third (weak effect) group of effects of the *α*_*s1*_*casein* genotypes, respectively. The column vectors ${\mathbf{t}}_{g1}$, ${\mathbf{t}}_{g2}$, and ${\mathbf{t}}_{g3}$ are the probabilities of an individual being in one of the above-mentioned three *α*_*s1*_*casein* genotype groups. For instance, ${\mathbf{t}}_{g1}$ is a vector computed as the sum of the probabilities that an individual carries genotypes (*AA*, *AB*, *AC*, *BB*, *BC*) of group 1. ${\mathbf{X}}_{2}$ is an incidence matrix relating observations to the probabilities of the fixed effects of each *α*_*s1*_*casein* genotype group. Here, ${\mathbf{e}}_{1}$ is assumed to be normally distributed as for Model (3). For Models (3) and (4), genomic breeding values were obtained by GBLUP using the blup90iod2 program [33].

Finally, we used a gene content approach [18, 35] to include the effect of *α*_*s1*_*casein* genotype in genomic (based on pedigree information and SNP genotypes) evaluations based on all female phenotypes. In this study, dairy goats carried six possible alleles at the *α*_*s1*_*casein* locus. The model used here i.e. Model (5a) was a seven-trait model including a gene content model for each allele (six gene contents) and the trait considered (here protein content):

5a $$\begin{aligned} {\mathbf{y}}_{1} &= {\mathbf{X}}_{1} {\varvec{\upbeta}}_{1} + {\mathbf{Z}}_{1} {\mathbf{u}}_{1} + {\mathbf{W}}_{1} {\mathbf{p}}_{1} + {\mathbf{e}}_{1} \hfill \\ {\mathbf{y}}_{\text{A}}& = {\varvec{\upmu}}_{\text{A}} + {\mathbf{Z}}_{\text{A}} {\mathbf{u}}_{\text{A}} + {\mathbf{e}}_{\text{A}} \hfill \\ {\mathbf{y}}_{\text{B}} &= {\varvec{\upmu}}_{\text{B}} + {\mathbf{Z}}_{\text{B}} {\mathbf{u}}_{\text{B}} + {\mathbf{e}}_{\text{B}} \hfill \\ {\mathbf{y}}_{\text{C}}& = {\varvec{\upmu}}_{\text{C}} + {\mathbf{Z}}_{\text{C}} {\mathbf{u}}_{\text{C}} + {\mathbf{e}}_{\text{C}} \hfill \\ {\mathbf{y}}_{\text{E}}& = {\varvec{\upmu}}_{\text{E}} + {\mathbf{Z}}_{\text{E}} {\mathbf{u}}_{\text{E}} + {\mathbf{e}}_{\text{E}} \hfill \\ {\mathbf{y}}_{\text{F}}& = {\varvec{\upmu}}_{\text{F}} + {\mathbf{Z}}_{\text{F}} {\mathbf{u}}_{\text{F}} + {\mathbf{e}}_{\text{F}} \hfill \\ {\mathbf{y}}_{\text{O}} &= {\varvec{\upmu}}_{\text{O}} + {\mathbf{Z}}_{\text{O}} {\mathbf{u}}_{\text{O}} + {\mathbf{e}}_{\text{O}} , \hfill \\ \end{aligned}$$

where $${\mathbf{y}}_{1}$$ is the vector of the 7,351,831 Alpine and Saanen records for protein content, $${\mathbf{X}}_{1}$$ is an incidence matrix relating observations to the same fixed effects $$\left( {{\varvec{\upbeta}}_{1} } \right)$$ as in Model (4), $${\mathbf{W}}_{1}$$ is an incidence matrix relating observations to permanent environmental effects $$\left( {{\mathbf{p}}_{1} } \right)$$ that are assumed to be normally distributed, $${\mathbf{Z}}_{1}$$ is an incidence matrix of the breeding genetic values $$\left( {{\mathbf{u}}_{1} } \right)$$ for the trait considered (i.e. protein content), $${\text{Var}}\left( {{\mathbf{u}}_{1} } \right) = {\mathbf{H}}\upsigma_{{{\text{u}}_{1} }}^{2}$$ and $${\mathbf{e}}_{1}$$ is the error term vector as in Model (4). Gene content vectors $${\mathbf{y}}_{A}$$, $${\mathbf{y}}_{B}$$, $${\mathbf{y}}_{C}$$, $${\mathbf{y}}_{E}$$, $${\mathbf{y}}_{F}$$ and $${\mathbf{y}}_{O}$$ are observed numbers of alleles for each of the six alleles, modeled as a mean fixed effect ($${\varvec{\upmu}}_{A}$$, $${\varvec{\upmu}}_{B}$$, $${\varvec{\upmu}}_{C}$$, $${\varvec{\upmu}}_{E}$$, $${\varvec{\upmu}}_{F}$$ or $${\varvec{\upmu}}_{O}$$ for alleles *A*, *B*, *C*, *E*, *F* or *O*, respectively), plus a random genetic effect ($${\mathbf{u}}_{A}$$, $${\mathbf{u}}_{B}$$, $${\mathbf{u}}_{C}$$, $${\mathbf{u}}_{E}$$, $${\mathbf{u}}_{F}$$ or $${\mathbf{u}}_{O}$$**)** representing the effect of alleles *A*, *B*, *C*, *E*, *F* or *O* respectively on protein content and random residual error ($${\mathbf{e}}_{A}$$, $${\mathbf{e}}_{B}$$, $${\mathbf{e}}_{C}$$, $${\mathbf{e}}_{E}$$, $${\mathbf{e}}_{F}$$ or $${\mathbf{e}}_{O}$$ for alleles *A*, *B*, C, *E*, *F* or *O*, respectively). In theory, $${\mathbf{e}}_{A}$$ to $${\mathbf{e}}_{O}$$ should be equal to 0, although, in practice, very small values are assigned to the residual variances, thus allowing some genotyping errors and the use of mixed model equations to estimate the breeding values [35]. $${\mathbf{Z}}_{A}$$, $${\mathbf{Z}}_{B}$$, $${\mathbf{Z}}_{C}$$, $${\mathbf{Z}}_{E}$$, $${\mathbf{Z}}_{F}$$, and $${\mathbf{Z}}_{O}$$ are incidence matrices relating observations to the genetic effect of gene content with $${\text{i}} \in \left\{ {{\text{A}},{\text{B}},{\text{C}},{\text{E}},{\text{F}},{\text{O}}} \right\}$$$${\text{Var}}\left( {{\mathbf{u}}_{\text{i}} } \right) = {\mathbf{H}}\upsigma_{{{\text{u}}_{\text{i}} }}^{2} = {\mathbf{H}}2p_{\text{i}} q_{\text{i}}$$, where $$p_{\text{i}}$$ is the allelic frequency of allele i at the *α*_*s1*_*casein* locus and $$q_{\text{i}} = 1 - p_{\text{i}}$$. The values of the vectors of gene content for individuals (males and females) that carried no copy of the considered allele, one copy, and two copies were 0, 1 and 2, respectively, and for ungenotyped animals, the value was set to missing. These vectors included the 6276 records (Table 1) for the animals genotyped at the *α*_*s1*_*casein* locus and 2,669,255 missing values for the ungenotyped females. In this model, genetic values were decomposed as a polygenic effect plus the effect of the *α*_*s1*_*casein* alleles. According to [35–38], the Model (5a) is equivalent to:

5b $$\begin{aligned} {\mathbf{y}}_{1} & = {\mathbf{X}}_{1} {\varvec{\upbeta}}_{1} + {\mathbf{z}}_{A} \upalpha_{A} + {\mathbf{z}}_{B} \upalpha_{B} + {\mathbf{z}}_{C} \upalpha_{C} + {\mathbf{z}}_{E} \upalpha_{E} \\ & \quad + {\mathbf{z}}_{F} \upalpha_{F} + {\mathbf{z}}_{O} \upalpha_{O} + {\mathbf{Z}}_{1} {\varvec{\upvarepsilon}} + {\mathbf{WZ}}_{1} {\mathbf{p}}_{1} + {\mathbf{e}}_{1} , \\ \end{aligned}$$

where ${\varvec{\upvarepsilon}}$ is the polygenic effect with ${\text{Var}}\left( {\varvec{\upvarepsilon}} \right) = {\mathbf{H}}\upsigma_{\upvarepsilon}^{2}$, scalars $\upalpha_{A}$, $\upalpha_{B}$, $\upalpha_{C}$, $\upalpha_{E}$, $\upalpha_{F}$ and $\upalpha_{O}$ are the effects of alleles *A*, *B*, *C*, *E*, *F* and *O*, respectively, and ${\mathbf{z}}_{A}$, ${\mathbf{z}}_{B}$, ${\mathbf{z}}_{C}$, ${\mathbf{z}}_{E}$, ${\mathbf{z}}_{F}$, and ${\mathbf{z}}_{O}$ are columns vectors, of size equal to the number of observations for copy number of alleles *A*, *B*, *C*, *E*, *F* and *O*, respectively. In order to include the effect of the alleles with registered gene content in the variance of the genetic value for protein content, (${\mathbf{u}}_{1}$ of Model 5a), the latter was derived as:

$${\text{Var}}\left( {{\mathbf{u}}_{1} } \right) = {\mathbf{H}}\upsigma_{{{\text{u}}_{1} }}^{2} = {\mathbf{H}}\left[ {\upsigma_{\upvarepsilon}^{2} + 2\mathop \sum \limits_{\text{i}} {\text{p}}_{\text{i}} {\text{q}}_{\text{i}} \upalpha_{\text{i}}^{2} - 2\mathop \sum \limits_{\text{i}} \mathop \sum \limits_{{{\text{j}} \ne {\text{i}}}} {\text{p}}_{\text{i}} {\text{q}}_{\text{j}} \upalpha_{\text{i}} \upalpha_{\text{j}} } \right],$$

for ${\text{i}}$ and ${\text{j}} \in \left\{ {A, B, C, E, F, O} \right\}$, where ${\text{p}}_{A}$, ${\text{p}}_{B}$, ${\text{p}}_{C}$, ${\text{p}}_{E}$, ${\text{p}}_{F}$ and ${\text{p}}_{O}$ correspond to the frequencies of alleles *A*, *B*, *C*, *E*, *F* and *O*, respectively, in the base population at the *α*_*s1*_*casein* locus and ${\text{q}}_{\text{i}} = 1 - {\text{p}}_{\text{i}}$. Covariances between genetic values $\left( {{\mathbf{u}}_{1} } \right)$ and genetic effects of gene content (${\mathbf{u}}_{A}$, ${\mathbf{u}}_{B}$, ${\mathbf{u}}_{C}$, ${\mathbf{u}}_{E}$, ${\mathbf{u}}_{F}$, ${\mathbf{u}}_{O}$) were modeled as:

$${\text{cov}}\left( \begin{array}{*{20}c} {{\text{u}}_{1} } \\ {{\text{u}}_{A} } \\ \cdots \\ {{\text{u}}_{O} } \\ \end{array} \right) = \left( \begin{array}{*{20}c} {{\mathbf{H}}{{\upsigma }}_{{{\text{u}}_{1} }}^{2} } & {{\mathbf{H}}{{\upsigma }}_{{{\text{u}}_{{1,A}} }} } & \ldots & {{\mathbf{H}}{{\upsigma }}_{{{\text{u}}_{{1,O}} }} } \\ {{\mathbf{H}}{{\upsigma }}_{{{\text{u}}_{{1,A}} }} } & {{\mathbf{H}}{{\upsigma }}_{{{\text{u}}_{A} }}^{2} } & \ldots & {{\mathbf{H}}{{\upsigma }}_{{{\text{u}}_{{A,O}} }} } \\ \cdots & \cdots & \cdots & \cdots \\ {{\mathbf{H}}{{\upsigma }}_{{{\text{u}}_{{1,O}} }} } & {{\mathbf{H}}{{\upsigma }}_{{{\text{u}}_{{O,A}} }} } & \ldots & {{\mathbf{H}}{{\upsigma }}_{{{\text{u}}_{O} }}^{2} } \\ \end{array} \right)$$

where ${\text{cov}}\left( {{\mathbf{u}}_{1} ,{\mathbf{u}}_{\text{i}} } \right) = 2{\mathbf{H}}{\text{p}}_{\text{i}} {\text{q}}_{\text{i}} \upalpha_{\text{i}} - 2{\mathbf{H}}\mathop \sum \nolimits_{\text{i}} \mathop \sum \nolimits_{{{\text{i}} \ne {\text{j}}}} {\text{p}}_{\text{i}} {\text{q}}_{\text{j}} \upalpha_{\text{j}}$ for ${\text{i}}$ and ${\text{j}} \in \left\{ {A, B, C, E, F, O} \right\}$ and covariances between effects of gene content were:

$${\text{cov}}\left( {\begin{array}{*{20}c} {\begin{array}{*{20}c} {{\mathbf{u}}_{A} } \\ \\ \cdots \\ \end{array} } \\ {{\mathbf{u}}_{O} } \\ \end{array} } \right) = 2{\mathbf{H}}\left( {\begin{array}{*{20}c} {\begin{array}{*{20}c} {{\text{p}}_{A} {\text{q}}_{A} } \\ { - {\text{p}}_{A} {\text{p}}_{\text{B}} } \\ \end{array} } \\ \ldots \\ { - {\text{p}}_{A} {\text{p}}_{O} } \\ \end{array} \begin{array}{*{20}c} {\begin{array}{*{20}c} { - {\text{p}}_{A} {\text{p}}_{B} } \\ {{\text{p}}_{B} {\text{q}}_{B} } \\ \end{array} } \\ \cdots \\ { - {\text{p}}_{B} {\text{p}}_{O} } \\ \end{array} \begin{array}{*{20}c} {\begin{array}{*{20}c} \ldots \\ \ldots \\ \end{array} } \\ \ldots \\ \ldots \\ \end{array} \begin{array}{*{20}c} {\begin{array}{*{20}c} { - {\text{p}}_{A} {\text{p}}_{O} } \\ { - {\text{p}}_{B} {\text{p}}_{O} } \\ \end{array} } \\ \ldots \\ {{\text{p}}_{O} {\text{q}}_{O} } \\ \end{array} } \right).$$

Equivalence between the multiple-trait model (Model 5b) and the univariate model (Model 5b) in which the effects of the allele are fitted as a covariable is described in detail in [35, 38]. Variance ($\upsigma_{{{\text{u}}_{1} }}^{2}$, $\upsigma_{{{\text{u}}_{A} }}^{2}$, …, $\upsigma_{{{\text{u}}_{O} }}^{2}$) and covariance ($\upsigma_{{{\text{u}}_{1,A} }}^{2}$, …, $\upsigma_{{{\text{u}}_{O,F} }}^{2}$) parameters from this model were estimated by the restricted maximum likelihood (REML) algorithm using remlf90 software [33].

The estimated genotypic effects obtained for Models (3), (4) and (5a) were used to include the effect of the *αs1 casein* genotype in genomic evaluations and were compared with estimates from a model that did not include any *α*_*s1*_*casein* effect, i.e. Model (6), which was exactly the same as Model (3):

6 $${\mathbf{y}}_{1} = {\mathbf{X}}_{1} {\varvec{\upbeta}}_{1} + {\mathbf{Z}}_{1} {\mathbf{u}}_{1} + {\mathbf{W}}_{1} {\mathbf{p}}_{1} + {\mathbf{e}}_{1} .$$

### Cross-validation analyses

In this study, cross-validation analyses consisted in splitting the population of the 823 males genotyped with the goat 50 K BeadChip into a training set of 677 males born before 2010 (with phenotypes of daughters recorded until January 2013), and a test set of 146 young males born between 2010 and 2011 and with no daughter by January 2013. The breeding values predicted for these 146 young males were compared with their DYD from the official genetic evaluation of January 2015, which were estimated by using a mean of 53 daughters per sire. The validation correlations consisted in Pearson correlations between the EBV or GEBV obtained in 2013 and the DYD obtained in 2015 for these 146 males. For Models (3) and (4), the GEBV were the sum of the estimated breeding values $\left( {{\mathbf{u}}_{1} } \right)$ and the estimated *α*_*s1*_*casein* effect (${\mathbf{s}}_{1}$ and ${\text{s}}_{g1} + {\text{s}}_{g2} + {\text{s}}_{g3} ,$ respectively). Differences between Pearson correlations obtained with or without including the effect of the *α*_*s1*_*casein* genotype in the models were analyzed using the Hotelling-Williams test [39].

## Results and discussion

### Frequencies of *α*_*s1*_*casein* genotypes in the French dairy goat population

**Fig. 1:**
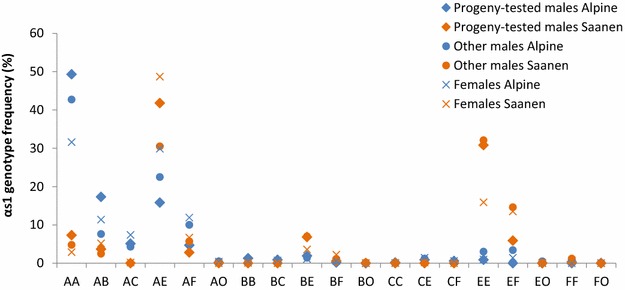
*α*_*s1*_ *casein* genotype frequencies in the French dairy goat population

The first objective of this study was to describe the current *α*_*s1*_*casein* allele frequencies in the French dairy goat population, which have not been reported since selection on the genotypes of males and dams of bucks was introduced [24]. The frequencies of *α*_*s1*_*casein* genotypes were estimated in six populations: 470 Alpine (1) and 353 Saanen (2) progeny-tested males that were genotyped with the goat 50 K BeadChip; 1442 Alpine (3) and 1062 Saanen (4) other males; and 1529 Alpine (5) and 1420 Saanen (6) dams of bucks (Fig. 1). The most frequent *α*_*s1*_*casein* genotypes in the French dairy goat population are *AA* and *AE* in the Alpine breed and *AE* and *EE* in the Saanen breed, which are present in more than 50 % of the animals. Most of the males carried the *AA* genotype, whereas most of the females carried the *AE* genotype. The distributions of the genotypes in progeny-tested males and in other males were similar. However, genotypes *AA* and *AB* in the Alpine and genotype *AE* in the Saanen breed were less frequent in the population of other males with more *AE* and *AF* genotypes in the Alpine and *EF* genotypes in the Saanen breed. These results differ from those reported for American Saanen and Alpine dairy goats for which the most frequent genotypes were *EF* and *FF* [40]. However, these frequencies are close to those found for French dairy goats born before 1989 with a predominance of allele *A* (41 and 18 % in Alpine and Saanen breeds, respectively) and allele *E* (54 and 26 % in the Saanen and Alpine breeds, respectively) [29]. The frequency of allele *A* reported in the current study was higher than that previously found in French dairy goats in the 1990s, which is due to genetic selection promoting allele *A* [24]. In our study, allele *C* was rather rare, with less than 5 % of the animals carrying this allele in the three subpopulations analyzed. These frequencies of allele *C* were close to those found in the French dairy goat population in 1989, which ranged from 1 to 2 % depending on the breed considered [29]. This is also consistent with studies on Mexican, Brazilian and American dairy goat breeds, for which no allele *C* was detected [40–42]. The frequencies of alleles *F* and *O* in a population of highly selected animals (progeny-tested males and dams of bucks) were lower than those reported in females born before 1989, i.e. 7.9 % for progeny-tested males and 18.9 % for dams of bucks versus 28 % in the Alpine and 24 % in the Saanen breeds [29]. This difference was also observed for animals that were not so strongly selected, i.e. the population of other males, possibly because their sires were males used for artificial insemination (AI) and selected on *α*_*s1*_*casein* genotype and their dams were females selected for high protein content [24, 25, 29]. In our study, the biggest differences in genotype frequency between Alpine and Saanen animals were observed for genotypes *AA* (49 % in Alpine vs. 7 % in Saanen progeny-tested males), *EE* (3 % in Alpine vs. 32 % in other Saanen males) and *AE* (49 % in Saanen vs. 30 % in Alpine females).These differences in frequencies were also observed in American dairy goats e.g. a frequency of 35.7 % for allele *E* in the Alpine versus 70.5 % in the Saanen breed [40]. In our study, alleles *A* and *E* were the most frequent alleles in the Alpine and Saanen breeds, respectively. Differences in frequencies for alleles *A* and *E* between the Alpine and Saanen breeds were previously reported by Martin and Leroux [24] in the French dairy goat population with a frequency of 14 % for allele *A* in the Alpine versus 7 % in the Saanen breed. Such a difference may be explained by of the fact that Saanen breeders are less involved in selecting animals on protein yield and content. In addition, the Saanen breed is more inbred than the Alpine breed [43]. Although bucks that carried allele *A*, *B* or *C* were preferentially chosen for AI, a program that aimed at managing inbreeding in the Saanen breed reduced the number of progeny-tested bucks carrying a strong allele (*A*, *B* or *C*) that could be used [29].

**Fig. 2:**
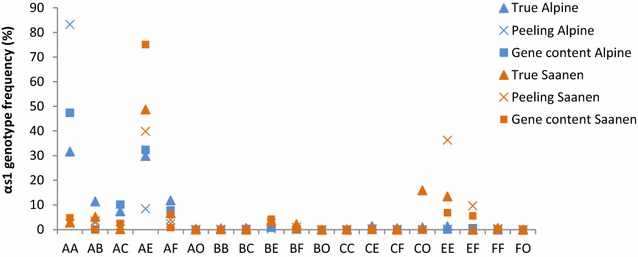
*α*_*s1*_ *casein* genotype frequencies for the dams of bucks. True Alpine and True Saanen are for genotyped females; Predicted Alpine and Predicted Saanen correspond to predicted frequencies of *α*_*s1*_ *casein * genotypes using peeling equations and the gene content approach

**Fig. 3:**
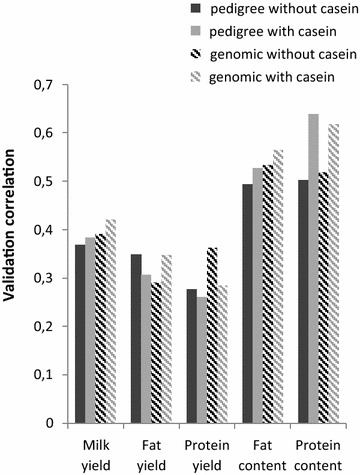
Validation correlations for the 146 validation males with or without *α*_*s1*_ *casein* genotype as fixed effect. Correlations between DYD in 2015 and GEBV in 2013. Pedigree without casein and Pedigree with casein correspond respectively to a model without and with α_s1_ casein effect using only pedigree to construct a relationship matrix. Genomic without casein and Genomic with casein correspond respectively to a model without or with fixed effect of *α*_*s1*_ *casein* genotype using pedigree and SNP genotype information to construct a relationship matrix

Next, we compared these *α*_*s1*_*casein* allele frequencies with those obtained by using predicted alleles or genotypes at the *α*_*s1*_*casein* locus by an iterative peeling method or gene content approach. Ideally, we should compare true and predicted genotypes using k-fold cross-validations. Given the small number of females (i.e. less than 500 animals) that were genotyped from each breed and each period (born before 2000, born between 2000 and 2007, and born after 2007) considered for the iterative peeling approach, we were only able to look at predicted genotype frequencies. To compare predicted genotypes of females estimated by an iterative peeling or gene content approach to the observed genotypes, we used genotype frequencies obtained for: (1) the 2949 genotyped females (named True Alpine and True Saanen in Fig. 2), (2) the 9303 females with a probability of at least 75 % of carrying a given genotype using predicted genotypes obtained by the iterative peeling approach (named Peeling Alpine and Peeling Saanen in Fig. 2), and (3) the same females as in (2) using the estimated genotypes (i.e. gene content of either one allele (if homozygous) or either two alleles (if heterozygous) from the six possible alleles that were most accurately estimated) obtained with the gene content approach (named Gene content Alpine and Gene content Saanen in Fig. 2). For the iterative peeling approach, frequencies were weighted by the probabilities of carrying genotypes that ranged from to 77 and 90 % for the given females. Thus, for genotype *AA*, we included all the females that had a probability of carrying genotype *AA* up to 75 %.

Differences between predicted genotype frequencies using the iterative peeling approach and real genotype frequencies were large (Fig. 2), especially for genotypes *AA* (32 % for the genotyped animals vs. 83 % with the iterative peeling method for the Alpine breed), *AE* (75 and 30 % for the genotyped animals vs. 40 and 8 %with the iterative peeling method for the Saanen and Alpine breed, respectively) and *EE* (13 % for the genotyped animals vs. 36 % with the iterative peeling method for the Alpine breed). The predicted frequencies were sometimes underestimated compared to the real frequencies (genotype *AE* for the Saanen breed) and sometimes overestimated (genotype *AA* for the Alpine breed and genotypes *EE* and *EF* for the Saanen breed). Genotype frequencies obtained with the gene content approach were closer to the real genotype frequencies than those obtained with the iterative peeling method, except for genotype *AE* in the Saanen breed. Previous reports showed no difference in the ability to predict genotypes between these two methods [18]. The differences that we observed between predicted genotype frequencies using the iterative peeling method and those using the gene content approach may be explained by the limited pedigree size, due to computational reasons, when using the peeling equations. Indeed, the gene content approach considered the whole pedigree for all individuals whereas the iterative peeling method considered three pedigrees (one for animals born before 2000, one for animals born between 2000 and 2007, and one for animals born after 2007). Because of this subdivision into three groups, the animals (parents or descendants) of one group were not taken into account to predict the genotypes of animals in either of the other two groups. We used an optimized iterative peeling method and it did not seem possible to improve the results by considering the whole pedigree. However, these differences could also be related to differences between real allele frequencies for dams of bucks and for other genotyped females. These differences can be large, as in our study, e.g. 31 % for alleles *F* and *O* in the Saanen breed [29] between females born from 1979 to 1987 and dams of bucks born from 1983 to 1989.

Inference of unknown genotypes is known to be a complex procedure because of the difficulty of obtaining a joint distribution of genotypes and complex traits [35]. In many previous studies, the predictions of unknown genotypes were based only on pedigree information [16, 18, 44, 45]. The extended gene content approach proposed by [35] allows to consider phenotype information which seems to improve genotype inference.

### Effect of the *α*_*s1*_*casein* genotype on traits selected in French dairy goats

One aim of this study was to identify the traits on which the *α*_*s1*_*casein* gene had a significant effect and to estimate the amount of variance explained by the *α*_*s1*_*casein* genotype. The effect of the genotype at the *α*_*s1*_*casein* locus, on the five milk production traits that are selected for in French dairy goats, was tested by using analysis of variance (Model 1) in: (1) a two-breed population (Alpine + Saanen), (2) a Saanen population and (3) an Alpine population. The results for the three groups were similar. The *α*_*s1*_*casein* genotype had a highly significant effect on protein and fat contents, with *R*-squared statistics between 0.11 and 0.20 for fat content and between 0.23 and 0.33 for protein content. It also had a significant effect on milk yield (*R*-squared statistics between 0.08 and 0.12). These results were consistent with those found previously in French dairy goats, except that no effect was detected on protein yield [23, 25]. Similarly, *α*_*s1*_*casein* haplotypes had a significant effect on protein content, fat yield and fat content in Norwegian dairy goats. The lack of any effect on protein yield can be explained by the highly negative genetic correlation (−0.28) between milk yield and protein content in Norwegian dairy goats [22]. This negative correlation that was estimated based on polygenic effects excluding the *α*_*s1*_*casein* gene effect appears to be strengthened from −0.42 to −0.48 [25] by taking *α*_*s1*_*casein* genotypes into account in the model.

Variance components estimated by considering the *α*_*s1*_*casein* genotype as a random effect in the model for milk yield, fat content and protein content are in Table 3. The amount of phenotypic variance explained by the effect of the *α*_*s1*_*casein* genotype was largest for the Alpine breed (for example, for milk yield: 6.1 % for Alpine vs. 3.3 % for Saanen). Polymorphism at the *α*_*s1*_*casein* locus explained between 24.4 % (Saanen) and 38.2 % (Alpine) of the variance for protein content and between 8.7 % (Saanen) and 18.2 % (Alpine) of the variance for fat content. These results for protein content are consistent with those obtained by [25] who reported a shift in polygenic heritability from 0.66 to 0.38 when the effect of the *α*_*s1*_*casein* genotype was included as fixed effect in the model.

**Table 3:** Amount of phenotypic variance explained by polygenic and *α*_*s1*_ *casein* effects for two-breed, Alpine, and Saanen populations

	Two-breed		Alpine		Saanen	
	*α*_*s1*_ *casein*	Polygenic	*α*_*s1*_ *casein*	Polygenic	*α*_*s1*_ *casein*	Polygenic
Milk yield	4.6	46.0	6.1	43.1	3.3	47.0
Fat content	13.7	54.0	18.2	56.5	8.7	43.7
Protein content	33.8	48.3	38.2	51.7	24.4	40.7

For protein content, the estimated effects of the *α*_*s1*_*casein* genotypes ranged from 3.7 for genotype *BC* to −0.9 g/kg for genotype *EF* in the Saanen breed (Table 2). *Casein* genotypes were grouped into three categories (Table 2): (1) genotypes with a strong effect on protein content up to 1.5 g/kg (genotypes *AA*, *AB*, *AC*, *BB* and *BC*), (2) genotypes with an intermediate effect on protein content between 0.5 and 1.5 g/kg (genotypes *AE*, *AF*, *BE*, *BF*, *CE* and *CF*) and (3) those with a weak effect on protein content up to 0.5 g/kg (genotypes *EE* and *EF*). Estimated effects on protein content were similar for the Alpine and Saanen breeds especially in the case of the genotypes *AE*, *AF* and *AA* with differences less than 0 g/kg. The largest differences between the two breeds were observed for genotypes *AB* (−0.8 g/kg in the Alpine compared to the Saanen breed) and *EE* (+0.9 g/kg in the Alpine compared to the Saanen breed). Although ranking of *α*_*s1*_*casein* genotypes according to their effect on protein content differed in the two breeds, the results for the three categories were similar. Considering the small observed differences between the estimated effects of *α*_*s1*_*casein* genotypes in the Saanen and Alpine breeds, the differences observed between the estimated variance components are likely explained essentially by differences in allele frequency.

### Including the effect of *α*_*s1*_*casein* genotype in analyses based on DYD

Given the small number of genotyped females (2949, Table 1) compared with the number of females with phenotypes (2,672,204, Table 1), the relevance of adding the *α*_*s1*_*casein* genotype as a fixed effect was first analyzed by using Model (2) based on male pseudo-performances (DYD). Figure 3 shows the correlations between the 2015 DYD and the (G)EBV predicted in 2013 for the 146 males born between 2010 and 2011 that were obtained by using four types of breeding values and two-breed populations i.e.: (1) only pedigree information in the relationship matrix without including the effect of the *α*_*s1*_*casein* genotype in the model [case (1) of Model 2]; (2) pedigree information in the relationship matrix and including the *α*_*s1*_*casein* genotype as a fixed effect [case (2) of Model 2]; (3) genomic (SNP genotypes) and pedigree information in the relationship matrix without including the effect of the α_s1_ casein genotype [case (3) of Model 2]; and (4) genomic (SNP genotypes) and pedigree information in the relationship matrix and including the *α*_*s1*_*casein* genotype as a fixed effect [case (4) of Model 2]. The validation correlations obtained when the *α*_*s1*_*casein* genotype was considered as a random effect were similar to those obtained when it was considered as a fixed effect (results not shown). Including the *α*_*s1*_*casein* genotype as a fixed effect in the genetic (based on pedigree information) or genomic (based on pedigree information and SNP genotypes) models significantly improved validation correlations for all traits except for fat and protein yields (Hotelling-Williams test, [39]). Using genomic information, instead of pedigree information only, improved validation correlations by 6 and 27 % for fat and protein contents, respectively. These results are consistent with those obtained for Lacaune meat sheep for which the *FeCL* gene was included in the genetic selection model for prolificacy. For French meat sheep, including the effect of this gene effect improved predictions of EBV, especially for the heterozygous females [46]. For fat and protein yields, for which no significant effect of the *α*_*s1*_*casein* genotype was found, adding the effect of the *α*_*s1*_*casein* genotype in either the genetic or genomic model decreased validation correlations by 12–21 % for fat and protein yield, respectively (non significant).

Regardless of adding information on the *α*_*s1*_*casein* genotype in the genetic evaluation (based on pedigree information) or in the genomic evaluation (based on pedigree information and SNP genotypes) improved validation correlations in a similar way for milk yield, fat and protein contents.

Validation correlations estimated separately, for each breed, for two-breed and single-breed training populations combining genomic and pedigree information, with the *α*_*s1*_*casein* genotype considered as a fixed effect in the model, are in Table 4. Results were slightly better when both Alpine and Saanen animals (two-breed population) were used than when only Saanen animals were included in the training population to predict Saanen validation males. Although the genetic distance between Alpine and Saanen breeds is small (<0.13 [47]), the two-breed training population performed less well for predicting milk yield, protein yield and protein content than the Alpine training population. However, the two-breed training population performed better for fat content and moderately better for fat yield than the Alpine training population. Differences between using a single-breed or a two-breed training population were greater for the Alpine breed, except for protein yield: from 1 % for fat yield to 49 % for fat content versus from 0 % for fat yield to 8 % for fat content for the Saanen breed. Higher validation correlations were obtained with the two-breed training population for all the traits in the Saanen breed and only for fat content in the Alpine breed. The two-breed training population performed better for the Saanen population probably because of the lower frequency of some genotypes at the *α*_*s1*_*casein* locus in the Saanen training population (genotypes *AA*, *AB* and *AC*, results not shown) compared with the Alpine population. These genotypes were rare in the Saanen training population but were more frequent in the Saanen validation population: 0.3 versus 3.9 %, (results not shown). Thus, their effects were not well predicted in the Saanen single-breed training population compared with the two-breed population.

**Table 4:** Validation correlations^a^ for validation males using *α*_*s1*_ *casein* genotype as fixed effect^b^ in two-breed, Saanen, and Alpine populations

	Single-breed Alpine	Single-breed Saanen	Two-breed Alpine	Two-breed Saanen
Milk yield	0.338	0.324	0.328	0.333
Fat yield	0.269	0.204	0.271	0.205
Protein yield	0.363	0.178	0.264	0.269
Fat content	0.232	0.360	0.346	0.390
Protein content	0.470	0.690	0.452	0.703

Single-breed Alpine and Single-breed Saanen correspond respectively to the Alpine training population used to predict the Alpine validation population and the Saanen training population used to predict the Saanen validation males

Two-breed Alpine and Two-breed Saanen correspond to the two-breed training population used to predict Alpine and Saanen animals, respectively

^a^Correlations between DYD in 2015 and GEBV in 2013

^b^ *α*_*s1*_ *casein* genotype was considered as a fixed effect

### Including probabilities of the *α*_*s1*_*casein* genotypes in one-step models

The relevance of adding information on the *α*_*s1*_*casein* genotype in the genomic evaluation based on single-step models was investigated by using the two approaches to estimate missing female genotypes. Table 5 shows validation correlations between the 2015 DYD and the GEBV predicted for protein content in 2013 for the males born between 2010 and 2011 in three cases: (1) two-breed training and validation populations, (2) Saanen population and (3) Alpine population with four of the six tested models (Models 3–6). Models (3) and (4) were based on probabilities of *α*_*s1*_*casein* genotypes that were obtained with the iterative peeling method for females with phenotypes. In Model (3) (“arbitrary probabilities”), the combination of probabilities for the 19 possible *α*_*s1*_*casein* genotypes was considered as a random effect (with 3553 levels). In Model (4) (“3 groups of probabilities”), three groups of probabilities were considered as described in “Methods” section. Model (5) (“gene content”) was based on a gene content approach [18, 35]. The results of these three models were compared with Model (6) i.e. a genomic single-step model without including the *α*_*s1*_*casein* genotype (“without *α*_*s1*_*casein*”) and are in Table 5. Validation correlations obtained with the “arbitrary probabilities” model were similar to those obtained using three groups of *α*_*s1*_*casein* fixed effects. In Table 5, the prediction ability of the gene content approach was higher than that of the other models (ranging from +4 to +14 %). This result is consistent with the study of Gengler et al. [18], in which a gene content approach, based on a biallelic marker, performed better than an iterative peeling method. It is also consistent with the differences in allele frequencies estimated on the genotypes that were predicted using the gene content approach, which were closer to the real frequencies than those obtained using the iterative peeling approach. Pearson correlations between predicted and known DYD for validation males were higher for the Saanen than for the Alpine breed with the three models used. This may be explained by the higher level of inbreeding and kinship in the Saanen breed [20]. Except for the Saanen breed, validation correlations using Models (3) and (4) that include the effect of the *α*_*s1*_*casein* genotype did not exceed those obtained with Model (6), which did not include the effect of the *α*_*s1*_*casein* genotype. Using the gene content approach, the validation correlations were slightly higher (from +4 % for the two-breed population to +14 % for the Saanen population) than those obtained by excluding the effect of the α_s1_*casein* genotype especially for single-breed evaluations (Alpine or Saanen). This result is consistent with the findings reported in a study on Canadian Holstein dairy cattle that took the *bovine transmembrane growth hormone receptor* genotype into account, with an increase from 0.3 to 0.5 % for somatic cell counts and milk yield, respectively [48]. The higher correlations obtained in our study could be due to the marked effect of the *α*_*s1*_*casein* genotype on protein content (between 24 and 38 % of the total phenotypic variance). The higher correlations obtained for the Saanen breed could be the result of the distribution of the *α*_*s1*_*casein* allele frequencies in the population. In the Saanen population, *α*_*s1*_*casein* genotypes *AE* and *EE* are carried by almost 65 % of the animals, which is nearly equivalent to a biallelic marker and much easier to predict.

**Table 5:** Validation correlations^a^ for the 146 validation males for models based on female phenotypes (one step) for protein content

	Arbitrary probabilities	Three probability groups	Gene content	Without *α*_*s1*_ *casein* information
Two-breed	0.66	0.65	0.75	0.72
Alpine	0.64	0.64	0.68	0.63
Saanen	0.84	0.84	0.86	0.75

The “arbitrary probabilities” model (Model 3) corresponds to the model using a combination of the 19 *α*_*s1*_ *casein* possible genotypes as a random effect

The “three probability groups” model (Model 4) corresponds to a model in which the effects of the three groups of possible genotypes (strong, moderate and weak effect on protein content) were considered as fixed effects

The “gene content” model (Model 5a) corresponds to a model using the gene content approach without using predicted probabilities of *α*_*s1*_ *casein* genotypes for females

The “without *α*_*s1*_ *casein* information” model (Model 6) corresponds to a model in which *α*_*s1*_ *casein* information was not considered

Two-breed results were obtained with both training and validation populations being two-breed (Alpine + Saanen) populations. Alpine and Saanen results were obtained with training and validation populations composed of either Alpine or Saanen animals, respectively

^a^Correlations between the 2015 DYD and the 2013 GEBV

This improvement in validation correlations by including an effect for the *α*_*s1*_*casein* genotype in the model was weaker than that found in the genomic evaluation based on the animals’ DYD. This may be due to the difficulty in predicting *α*_*s1*_*casein* genotypes for non-genotyped females, especially since a large proportion (40 % in French dairy goats) of the females came from unknown parents. Even in a subpopulation of females with known parents and for which one-third of the females have at least one genotyped parent (results not shown), improvements in validation correlations were smaller than expected from the results of the analyses based on DYD. Predicting *α*_*s1*_*casein* genotypes for non-genotyped animals even when using the gene content approach is particularly difficult in this case because of the large number of alleles considered. Even for individuals with one genotyped parent, the number of possible genotypes was too large to accurately predict their possible *α*_*s1*_*casein* genotypes. In addition, several *α*_*s1*_*casein* alleles (*A*, *B* and *C*) have the same effect on some phenotypes (protein content, fat content or milk yield), thus calculating predictions with the gene content approach is even more difficult. One solution could be to consider gene content of groups of alleles that have the same effects on the trait.

## Conclusions

This study set out to determine how to include the effect of the *α*_*s1*_*casein* major gene, which has a complex polymorphism, in the genetic evaluation of French dairy goats. First, genotype frequencies in the Alpine and Saanen population showed differences between males and females and between Alpine and Saanen animals. The *α*_*s1*_*casein* genotype had an effect on several production traits in French dairy goats: milk yield, fat content and protein content with a large amount of phenotypic variance explained by the *α*_*s1*_*casein* genotype for protein content. Including an effect of the *α*_*s1*_*casein* genotype in a genetic evaluation that is based on animals with known *α*_*s1*_*casein* genotypes (analyses based on DYD), improved accuracy even when SNP genotypes had been taken into account. Including the effect of the *α*_*s1*_*casein* genotype in genetic evaluations that are based on female phenotypes and genotype probabilities yielded a lower accuracy than the approach that was based on gene content. Finally, improvement in validation correlations, by including the *α*_*s1*_*casein* effect in the models, was greater for genetic evaluations that were based on animals genotyped at the *α*_*s1*_*casein* gene. However, a smaller improvement was obtained for genetic evaluations that were based on ungenotyped animals at the *α*_*s1*_*casein* gene, due to the difficulty in predicting multi-allelic genotypes for this gene.

## References

[1] Palhiere I, Brochard M, Moazami-Goudarzi K, Laloë D, Amigues Y, Bed’hom B (2008). Impact of strong selection for the PrP major gene on genetic variability of four French sheep breeds. Genet Sel Evol.

[2] Nagy B, Fésüs L, Safar L. Breeding for scrapie resistance and control strategies in Hungary. In Proceedings of the 56th European Federation for Animal Science meeting: 5–8 June 2005; Uppsala. 2005.

[3] Meuwissen THE, Hayes BJ, Goddard ME (2001). Prediction of total genetic value using genome-wide dense marker maps. Genetics.

[4] Nicholas FW (2006). Discovery, validation and delivery of DNA markers. Aust J Exp Agric.

[5] Ducrocq V, Croiseau P, Baur A, Saintilan R, Fritz S, Boichard D. Genomic evaluations using QTL information. In: Proceedings of the 10th world congress on genetics applied to livestock production: 17–22 August 2014; Vancouver. 2014.

[6] Odegard J, Sonesson AK, Yazdi MH, Meuwissen TH (2009). Introgression of a major QTL from an inferior into a superior population using genomic selection. Genet Sel Evol.

[7] Hayes BJ, Bowman PJ, Chamberlain AC, Verbyla K, Goddard ME (2009). Accuracy of genomic breeding values in multi-breed dairy cattle populations. Genet Sel Evol.

[8] Verbyla KL, Hayes BJ, Bowman PJ, Goddard ME (2009). Accuracy of genomic selection using stochastic search variable selection in Australian Holstein Friesian dairy cattle. Genet Res.

[9] De Roos APW, Schrooten C, Druet T (2011). Genomic breeding value estimation using genetic markers, inferred ancestral haplotypes, and the genomic relationship matrix. J Dairy Sci.

[10] Calus MPL, de Roos APW, Veerkamp RF (2008). Accuracy of genomic selection using different methods to define haplotypes. Genetics.

[11] Edriss V, Fernando RL, Su G, Lund MS, Guldbrandtsen B (2013). The effect of using genealogy-based haplotypes for genomic prediction. Genet Sel Evol.

[12] Croiseau P, Fouilloux M-N, Jonas D, Fritz S, Baur A, Ducrocq V, Phocas F, Boichard D. Extension to haplotypes of genomic evaluation algorithms. In: Proceedings of the 10th world congress on genetics applied to livestock production: 17–22 August 2014; Vancouver. 2014.

[13] Boichard D, Fritz S, Rossignol MN, Boscher MY, Malafosse A, Colleau JJ. Implementation of marker-assisted selection in French dairy cattle. In: Proceedings of the 7th world congress on genetics applied to livestock production: 19–23 August 2002; Montpellier. 2002.

[14] VanRaden PM (2008). Efficient methods to compute genomic predictions. J Dairy Sci.

[15] Christensen OF, Lund MS (2010). Genomic prediction when some animals are not genotyped. Genet Sel Evol.

[16] Vitezica ZG, Elsen JM, Rupp R, Diaz C (2005). Using genotype probabilities in survival analysis: a scrapie case. Genet Sel Evol.

[17] Fernando RL, Stricker C, Elston RC (1993). An efficient algorithm to compute the posterior genotypic distribution for every member of a pedigree without loops. Theor Appl Genet.

[18] Gengler N, Mayeres P, Szydlowski M (2007). A simple method to approximate gene content in large pedigree populations: application to the myostatin gene in dual-purpose Belgian Blue cattle. Animal.

[19] Carillier C, Larroque H, Robert-Granié C (2014). Comparison of joint versus purebred genomic evaluation in the French multi-breed dairy goat population. Genet Sel Evol.

[20] Carillier C, Larroque H, Palhière I, Clément V, Rupp R, Robert-Granié C (2013). A first step toward genomic selection in the multi-breed French dairy goat population. J Dairy Sci.

[21] Grosclaude F, Mahé MF, Brignon G, Di Stasio L, Jeunet R (1987). A Mendelian polymorphism underlying quantitative variations of goat αs1-casein. Genet Sel Evol.

[22] Hayes B, Hagesæther N, Ådnøy T, Pellerud G, Berg PR, Lien S (2006). Effects on production traits of haplotypes among casein genes in Norwegian goats and evidence for a site of preferential recombination. Genetics.

[23] Mahé MF, Manfredi E, Ricordeau G, Piacère A, Grosclaude F (1993). Effets du polymorphisme de la caséine αs1 caprine sur les performances laitières: analyse intradescendance de boucs de race Alpine. Genet Sel Evol.

[24] Martin P, Leroux C. Le gène caprin spécifiant la caséine αs1: un suspect tout désigné aux effets aussi multiples qu’inattendus. Prod Anim. 2000; Special issue:125–132.

[25] Barbieri ME, Manfredi E, Elsen JM, Ricordeau G, Bouillon J, Grosclaude F (1995). Effects of the αs1-casein locus on dairy performances and genetic parameters of Alpine goats. Genet Sel Evol.

[26] Larroque H, Astruc JM, Barbat A, Barillet F, Boichard D, Bonaïti B, et al. National genetic evaluations in dairy sheep and goats in France. In: Proceedings of the 62th European federation of animal science (EAAP): 29 August—2 September 2011; Stavanger. 2011.

[27] Leroux C, Le Provost F, Petit E, Bernard L, Chilliard Y, Martin P (2003). Real-time RT-PCR and cDNA macroarray to study the impact of the genetic polymorphism at the alphas1-casein locus on the expression of genes in the goat mammary gland during lactation. Reprod Nutr Dev.

[28] Selvaggi M, Laudadio V, Dario C, Tufarelli V (2014). Major proteins in goat milk: an updated overview on genetic variability. Mol Biol Rep.

[29] Grosclaude F, Ricordeau G, Martin P, Remeuf F, Vassal L, Bouillon J (1994). From gene to cheese: the caprine αs1-casein polymorphism, its effects and its evolution. Prod Anim.

[30] Legarra A, Aguilar I, Misztal I (2009). A relationship matrix including full pedigree and genomic information. J Dairy Sci.

[31] Tosser-Klopp G, Bardou P, Bouchez O, Cabau C, Crooijmans R, Dong Y (2014). Design and characterization of a 52 K SNP chip for goats. PLoS One.

[32] Fikse WF, Banos G (2001). Weighting factors of sire daughter information in international genetic evaluations. J Dairy Sci.

[33] Misztal I, Tsuruta S, Strabel T, Auvray B, Druet T, Lee DH. BLUPF90 and related programs (BGF90). In: Proceedings of the 7th world congress on genetics applied to livestock production: 19–23 August 2002; Montpellier. 2002.

[34] Bélichon S, Manfredi E, Piacère A (1999). Genetic parameters of dairy traits in the Alpine and Saanen goat breeds. Genet Sel Evol.

[35] Legarra A, Vitezica ZG (2015). Genetic evaluation with major genes and polygenic inheritance when some animals are not genotyped using gene content multiple-trait BLUP. Genet Sel Evol.

[36] Yu J, Pressoir G, Briggs WH, Vroh Bi I, Yamasaki M, Doebley JF (2006). A unified mixed-model method for association mapping that accounts for multiple levels of relatedness. Nat Genet.

[37] Kang HM, Sul JH, Service SK, Zaitlen NA, Kong S, Freimer NB (2010). Variance component model to account for sample structure in genome-wide association studies. Nat Genet.

[38] Kennedy BW, Quinton M, van Arendonk AJ (1992). Estimation of effects of single genes on quantitative traits. J Anim Sci.

[39] Williams EJ (1959). The comparison of regression variables. J R Stat Soc Ser B Stat Methodol.

[40] Maga EA, Daftari P, Kültz D, Penedo MCT (2009). Prevalence of alphas1-casein genotypes in American dairy goats. J Anim Sci.

[41] Torres-Vazquez JA, Vazquez Flores F, Montaldo HH, Ulloa-Arvizu R, Valencia Posadas M, Gayosso Vazquez A (2008). Genetic polymorphism of the αs1-casein locus in five populations of goats from Mexico. Electron J Biotechonol.

[42] Soares MAM, Rodrigues MT, Mognol GP, Ribeiro LdFC, Silva JLdC, Brancalhão RMC (2009). Polymorphism of alpha s1-casein gene in a dairy goat herd in the southeastern region of Brazil. R Bras Zootec..

[43] Gipson T. Preliminary observations: Inbreeding in dairy goats and its effects on milk production. In: Proceedings of the 17th annual Goat Field Day: 26–27 April 2002; Langston. 2002.

[44] Mulder HA, Lidauer MH, Vilkki JH, Strandén I, Veerkamp R (2011). Marker-assisted breeding value estimation for mastitis resistance in Finnish Ayrshire cattle. J Dairy Sci.

[45] Fernando RL, Grossman M (1989). Marker assisted selection using best linear unbiased prediction. Genet Sel Evol.

[46] Martin P, Raoul J, Bodin L (2014). Effects of the *FecL* major gene in the Lacaune meat sheep population. Genet Sel Evol.

[47] de Araujo AM, Guimaraess SEF, Machado TMM, Lopes PS, Pereira CS, da Silva FLR (2006). Genetic diversity between herds of Alpine and Saanen dairy goats and the naturalized Brazilian Moxoto breed. Genet Mol Biol..

[48] Gengler N, Abras S, Verkenne C, Vanderick S, Szydlowski M, Renaville R (2008). Accuracy of prediction of gene content in large animal populations and its use for candidate gene detection and genetic evaluation. J Dairy Sci.

